# Have public attitudes towards people with mental health conditions shifted in Singapore? Results from the mental health literacy study

**DOI:** 10.1007/s00127-026-03067-7

**Published:** 2026-02-24

**Authors:** Savita Gunasekaran, Eng Hong Tay, Shazana Shahwan, Yoke Boon Tan, Wei Jie Ong, Bernard Chin Wee Tan, Saleha Shafie, Porsche Poh, Edimansyah Abdin, Siow Ann Chong, Mythily Subramaniam

**Affiliations:** 1 Present Address: Research Division, Institute of Mental Health, 10 Buangkok View, Singapore, 539747 Singapore; 2 Silver Ribbon Singapore, Singapore, 550208 Singapore; 3 Saw Swee Hock School of Public Health, National University of Singapore, Singapore, 117549 Singapore

**Keywords:** Mental health literacy, Public attitudes, Multi-ethnic population, Population-based study, Singapore

## Abstract

**Background:**

Public attitudes towards people with mental illnesses (PMI) shape their inclusion in society. In recent years, Singapore has introduced several nationwide mental health initiatives, including destigmatisation efforts. The present study explored the differences in attitudes among the Singapore public in 2023 compared to 2015, and the sociodemographic correlates of attitudes in 2023.

**Methods:**

Nationwide studies were conducted with residents in 2014–2015 (*N* = 3006) and 2022–2023 (*N* = 4195). Attitudes towards PMI included four factors: (1) social distancing, (2) tolerance/support for community care, (3) social restrictiveness, and (4) prejudice and misconception. Lower scores indicate better attitudes except for tolerance/support, where higher scores indicate better attitudes. Linear regression analysis was conducted to evaluate the differences in attitudes between 2015 and 2023, and multivariate linear regression was conducted to examine the sociodemographic correlates for each factor score.

**Results:**

Respondents scored significantly better in 2023 as compared to 2015 for all factors (*p* < 0.001): social distancing (7.19 vs. 8.07), tolerance/support (15.23 vs. 14.81), social restrictiveness (6.22 vs. 7.21), and prejudice and misconception (14.06 vs. 15.36). Age, gender, marital status, ethnicity, educational levels and income levels were associated with attitudes in various domains.

**Discussion & Conclusion:**

Improved public attitudes indicate a less stigmatising society. However, certain demographics continue to display poorer attitudes, underscoring the need for targeted interventions. Future efforts should integrate mental health promotion with primary care settings, engage community leaders, and provide opportunities for contact with PMI. Further research could also examine how factors such as prior contact with PMI shape these attitudes.

## Introduction

Attitudes towards people with mental illnesses (PMI) refer to personal beliefs regarding PMI and how they ought to be treated [1, 2, 3]. These attitudes can range from positive ones, including acceptance and tolerance, to negative ones, including fear and unwillingness to engage in close relationships [4]. The notion that public attitudes play a salient role in the recovery and quality of life of PMI has been consistently cited in the literature [5]. Stigmatising attitudes deter help-seeking behaviours due to fear of being labelled with a diagnosis [6]. Previous studies have also linked national levels of public stigma to discrimination faced by PMI and their willingness to seek treatment [5, 7, 8]. Additionally, the internalisation of negative public attitudes can result in a reduction of self-esteem, self-efficacy and sense of self-worth, which might, in turn, affect PMI’s mental health further [3, 9]. In contrast, support and acceptance from the public can facilitate help-seeking and improve communication between PMI and care providers and loved ones [10]. It can also create better opportunities for PMI, for example, in terms of employment [11].

Sociocultural influences contribute to shaping the emergence and persistence of negative attitudes, as well as the effectiveness of anti-stigma efforts. Evidence indicates that the nature and degree of stigma in Asian societies differ from those observed in Western contexts [12,13]. Asian societies typically have shared sociocultural features such as collectivism, emphasis on social harmony, and stigma linked to “loss of face”, which strongly influence public attitudes towards PMI [3]. Furthermore, in many Asian societies, mental illnesses are often moralised, with PMIs perceived as having poorer character due to cultural and religious beliefs, contributing to stronger stigma [14,15,16,17,18]. Beyond that, religious interpretations such as notions of sin, possession or karma reinforce moral judgements towards PMI [18,19,20,21,22,23]. Compared to many other Asian countries, Singapore has a more developed mental healthcare system and comparatively more positive attitudes [12]. However, despite the availability of well-established services, Singapore continues to face a substantial treatment gap, as many individuals avoid help-seeking due to stigma [24]. A local epidemiological study revealed that, despite general positive attitudes, considerable negative stigma towards PMI persists [25]. While most respondents reported willingness to interact socially with PMI, notable proportions indicated discomfort with closer contact, such as living next door or working closely with a PMI [25]. A recent qualitative study on daily encounters with stigma reported that PMI’s face substantial public stigma, including contemptuous treatment and social exclusion [26]. These respondents also shared that they were often perceived as dangerous, unpredictable, and inferior, with their condition being viewed as a character flaw [26].

In 2015, a nationwide study investigating mental health literacy and stigma among the general population in Singapore, titled *Mind Matters 2015*, was launched. The study utilized a scale that measures public attitudes towards PMI in the Singapore general population. The basis was that, although improving mental health knowledge was seen among the public, stigmatisation and discrimination remained persistently the same [27, 28]. Additionally, public attitudes, which include positive aspects such as social support and acceptance, had been a largely neglected research area compared to stigma, which only encompasses negative attitudes [4]. This highlighted a need for a scale to measure public attitudes in Singapore. Yuan and colleagues (2016) adopted the UK-based Attitudes to Mental Illness questionnaire to develop a locally validated scale to measure attitudes [29]. The adapted scale consisted of four factors: (1) social distancing, (2) tolerance/support for community care, (3) social restrictiveness, and (4) prejudice and misconception, elucidating the multidimensionality of public attitudes towards PMI in Singapore [4]. The study revealed the presence of negative attitudes towards PMI, particularly in certain groups of people – older adults, males, and those with lower socioeconomic status [4]. Those of Chinese ethnicity also showed more negative attitudes than those belonging to Indian and Malay ethnic groups (except for prejudice and misconception) [4]. These findings emphasised the importance of culturally relevant local destigmatisation efforts [4]. As this study was conducted more than eight years ago, up-to-date evidence of attitudes and associated correlates is needed to understand current patterns.

Since 2015, an increasing number of initiatives have been introduced in Singapore with the aim of normalising conversations surrounding mental health, decreasing stigma, and increasing inclusivity of PMI. A 2023 news report noted that mental health professionals observed a rise in mental health discussions since the onset of the COVID-19 pandemic [30]. A narrative review involving eight psychiatrists across China, India, Japan, Nepal, Thailand, the Philippines, Lebanon, and Singapore highlighted shared sociocultural factors and underlying stigmatising beliefs that contribute to negative perceptions of mental illnesses in their respective nations [31]. Across the nations discussed, common themes included the moralisation of mental illness as a sign of weak character, the influence of collectivist values that prioritise family honour and social harmony, and the persistence of supernatural or religious explanations [15]. Encouragingly, the review also showed that more recent destigmatising efforts have been steered in these countries, and some positive shifts in attitudes were evident [31]. However, as these findings were largely based on the perspectives of mental health professionals, investigating public attitudes directly is essential to substantiate these claims.

With the introduction of new mental health initiatives and increasing conversations about mental health in recent years, one would expect changes in public attitudes. Therefore, it would be timely to explore these changes within the nation to enable policymakers and service providers to not only evaluate the impact of various mental health initiatives implemented, but also to further improve and strategize on these initiatives and identify emerging trends that may be significant.

As such, the present study had two main objectives: (1) to examine the differences, if any, in the four factors of public attitudes towards PMI between 2015 and 2023, and (2) to identify sociodemographic correlates of each attitude factor in 2023. It was hypothesised that attitudes towards PMI would improve across all four domains in 2023 compared to 2015. Additionally, an exploratory approach was adopted to examine which of the sociodemographic characteristics, specifically age, gender, marital status, employment status, education level, and income, were associated with public attitudes towards PMI in 2023.

## Methods

### Sample

Data were from two nationwide cross-sectional studies of mental health literacy conducted in Singapore, the first from March 2014 to April 2015 (*Mind Matters 2015*) and the second from September 2022 to February 2024 (*Mind Matters 2023*). Both studies adopted a disproportionate stratified sampling design with 12 strata by age (18–34, 35–49, 50–65) and ethnicity groups (Chinese, Malay, Indian, and other ethnic groups). A probability sample was randomly selected through a registry containing names and sociodemographic data such as age, ethnicity and household address of Singapore residents. The inclusion criteria for both studies were (1) Singapore residents, (2) aged between 18 and 65 years old, and (3) living in Singapore during the study period. The exclusion criteria included individuals who were (1) not in Singapore during the study period, (2) unable to be contacted due to incomplete and incorrect residential addresses, (3) unable to comprehend the interview in one of the four specified local languages (English, Chinese, Malay or Tamil), and/or (4) physically and/or mentally unable to complete the interview. Before the interview, respondents were screened for their ability to provide consent and participate meaningfully. Mental capacity was determined based on their understanding of the study purpose and ability to communicate coherent responses. Physical ability was assessed by confirming that the participant could engage in the interview without undue distress or fatigue. In some cases, caregivers informed the interviewers that the selected respondent was physically or mentally unable to participate due to reasons such as being weak from a terminal illness or having an intellectual disability. More detailed information on the sampling strategy is found in the paper by Chong et al. [32] and Tan et al. [33]. 3006 (response rate of 71.1%), and 4195 (response rate of 62.3%) respondents participated in 2015 and 2023, respectively.

Overall, respondents had an average age of 40.9 (SD = 13.4) in 2015 and 43.2 (SD = 13.5) in 2023. In both studies, there was an almost equal percentage of males and females (2015 males = 50.9%, 2023 males = 48.8%). The weighted ethnicity distribution was reflective of the Singapore population, with the majority being Chinese (2015 = 74.7%, 2023 = 73.8%), followed by Malays (2015 = 12.8%, 2023 = 13.2%) and Indians (2015 = 9.1%, 2023 = 9.2%). Most respondents were married (2015 = 64.0%, 2023 = 60.3%) and/or employed (2015 = 77.6%, 2023 = 80.9%). The education level of the respondents leaned towards moderately high, with 31.3% having a diploma and 29.6% having a bachelor’s degree and above in 2015, and 30.9% having a diploma and 40.3% having a bachelor’s degree in 2023, respectively. Most respondents had a monthly salary lower than SGD 6000 (2015 = 86.9%, 2023 = 76.4%). Further details about the samples are presented in Table 1.

**Table 1:** Sociodemographic correlates for Mind Matters 2015 and Mind Matters 2023

	*Mind Matters 2015 (n = 3006)*		*Mind Matters 2023 (n = 4195)*	
	Weighted %	Unweighted *n*	Weighted %	Unweighted *n*
**Age groups**				
18–34	34.38	1152	31.65	1377
35–49	35.17	896	31.64	1315
50–67	30.45	958	36.71	1503
**Gender**				
Female	49.07	1506	51.17	2149
Male	50.93	1500	48.83	2046
**Ethnicity**				
Chinese	74.74	1034	73.84	1150
Malay	12.84	977	13.29	1347
Indian	9.11	963	9.25	1322
**Marital status**				
Never Married	31.41	927	32.86	1278
Currently married	64.01	1916	60.32	2630
Separated/ Divorced/ Widowed	4.58	162	6.81	287
**Highest education**				
Primary and below	13.39	431	7.26	318
Secondary/ O Levels/ N Levels	25.76	820	21.56	976
A Level/ Polytechnic Diploma/ Other Diploma	31.28	999	30.85	1454
Bachelor’s Degree and above	29.57	756	40.33	1447
**Employment status**				
Currently employed	77.64	2227	80.93	3322
Unemployed	3.92	120	4.04	179
Economically inactive	18.43	659	15.02	694
Monthly personal income*				
Below SGD2,000 (USD1489)	40.49	1346	30.72	1433
SGD2000 to SGD5999 (USD1489 to USD 4466)	46.44	1162	45.65	1926
SGD6000 and above (USD4468 and above)	13.07	294	23.62	776

*USD conversations were calculated using the average 2023 exchange rate

### Measurements

**Fig. 1:**
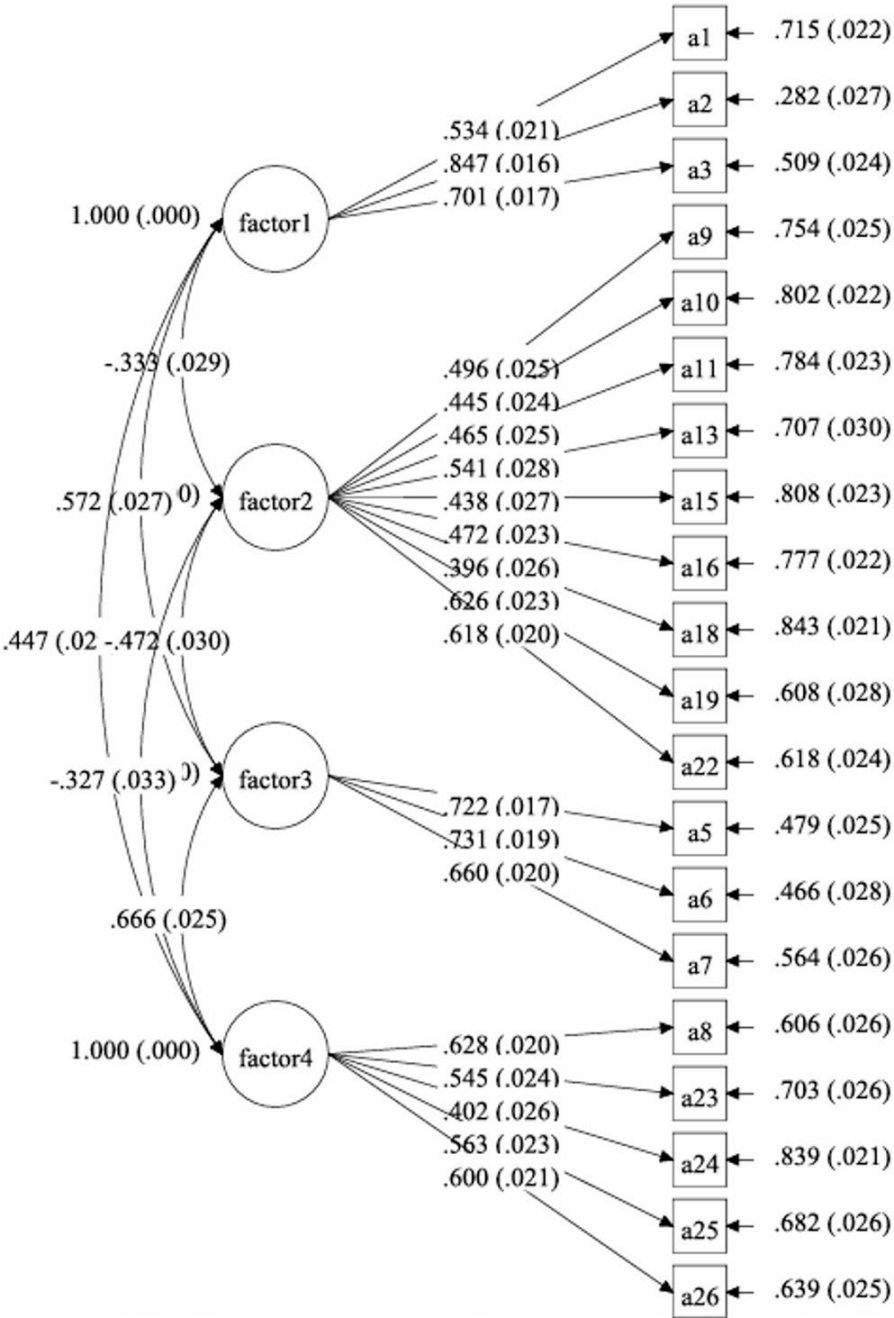
Factor loadings of CFA analysis on ATPMI for 2023 dataset (*n* = 4195). *Note*: factor1: Social distancing, factor2: Tolerance/support for community care, factor3: Social restrictiveness, factor4: Prejudice and misconception

Attitudes towards people with mental illnesses (ATPMI) is a 26-item scale adapted from a tool originally developed by the UK Department for Health [4, 29]. The scale was translated into three other languages (Chinese, Malay, Tamil) by professional translators. The translations were reviewed by bilingual researchers from the study team to ensure linguistic accuracy and cultural appropriateness. Cognitive interviews were conducted to assess the clarity and cultural relevance of the questionnaire items for all languages, providing content validation to ensure that questions were interpreted as intended. The scale was validated through a factor analysis during Mind Matters 2015, deriving a four-factor structure [4]. The first factor, ‘Social distancing’, consists of 3 items (score range: 3 to 15) and measures people’s intention to distance themselves from PMI in their community. The second factor, ‘Tolerance/support for community care’ consists of 9 items (score range: 9 to 45). It measures public’s understanding and tolerance towards PMI and their views towards integrating mental health services into the community. The third factor, ‘social restrictiveness’, consists of 3 items (score range: 3 to 15). It measures public’s attitudes about limiting the societal roles and responsibilities of PMI. The last factor relates to public’s ‘prejudice and misconception’ over mental health conditions and comprises 5 items (score range: 5 to 25). Items were rated on a 5-point Likert scale ranging from ‘1 = strongly agree’ to ‘5 = strongly disagree’. Lower scores indicate better attitudes for ‘social distancing’, ‘social restrictiveness’, and ‘prejudice and misconception’, and poorer attitudes for ‘tolerance/support for community care’. For the present study, Confirmatory Factor Analysis (CFA) was conducted to test the model fit of this 4-factor model among the 2023 dataset, which resulted in a satisfactory model fit. The individual factor loadings are depicted in Fig. 1. The internal consistency reliability statistics for the four factors were 0.740, 0.748, 0.755, and 0.699, respectively.

Sociodemographic variables, namely age, gender, ethnicity, marital status, education level, employment status and personal monthly income were also collected.

### Procedures

The study was conducted through face-to-face interviews by lay interviewers in one of the four languages. 40 interviewers from the contracted survey company for the study were trained by the IMH study team on recruitment and data collection. Interviewers were familiarised with the interview materials and consent-taking procedures, received background information on mental health literacy in Singapore, and participated in roleplay sessions with the study team to practice handling potential scenarios during data collection. Bilingual training was also provided for interviewers who would be administering the questionnaire in more than one language. The training concluded with an evaluation, in which each interviewer was individually assessed by a study team member through a simulated interview, administering the questionnaire. Only interviewers who successfully passed the evaluation proceeded to data collection. For quality assurance, a study team member accompanied each interviewer during their initial data collection sessions to observe their performance and provide feedback.

The field work included recruitment, data collection and quality control. Replicates, each containing 500–1300 randomly selected residents’ names, addresses and basic sociodemographic information, were sent directly to the survey company and distributed among interviewers. Invitation letters detailing the purpose and procedures of the study were mailed to the potential respondents at least two weeks before the interviewers’ visits.

Recruitment involved making visits to the potential respondents’ addresses. When contact was made with a potential respondent, interviewers were to confirm their details and eligibility and explain the study. If contact was made with the residents of the household who confirmed that the potential participant resided at the address, one or the combination of the following follow-up actions were taken: (1) repeated visits, (2) leaving a While You Were Out (WYWO) card for the participant to contact them, or (3) phone call (if the resident was willing to provide a contact number). Respondents who agreed to take part could do so on the spot or schedule the interview on another day. WYWO cards were left on potential respondents’ doorsteps if no one was at home. Visits ceased only if the interviewers received the selected resident’s refusal to partake in the study, confirmed ineligibility of the resident, or if there was no available information about them from the household. Common reasons for refusal included lack of time, perceived inconvenience, privacy or confidentiality concerns, and disinterest in the study topic. Up to 10 contact attempts had to be made before classifying a particular resident as a “non-responder”.

Interviews were conducted at respondents’ preferred time and location. Before data collection, written informed consent was obtained from all respondents and from parents/guardians of respondents who were below 21 years old. Data was collected through a tablet, and the questionnaire was displayed using a dual-language screen. This allowed bilingual interviewers to translate phrases and words as required by the respondent in a consistent manner. Interviewers read aloud every question and entered the responses. A respondent booklet was given to the respondent for their reference, where applicable. Respondents received an inconvenience fee of SGD 40 upon completion. Both studies were approved by the National Healthcare Group Domain Specific Review Board in Singapore.

### Data analysis

All estimates were weighted to adjust for over-sampling and post-stratified for age and ethnicity distributions to represent the Singapore resident population for both studies. Linear regression analysis was conducted to evaluate the differences in attitudes between 2015 and 2023, adjusting for sociodemographic variables (i.e. age, gender, ethnicity, marital status, education level, employment status and personal income). Multivariable linear regression was conducted to examine the sociodemographic correlates for each of the ATPMI factor scores. An alpha value below 0.05 was considered statistically significant. The descriptive and multivariate linear regression analyses were conducted using STATA SE Version 15 and Mplus version 8.8.

## Results

### Comparison of mean scores of ATPMI between 2015 and 2023

Lower social distancing, social restrictiveness, and prejudice and misconception scores and higher tolerance/support for community care scores indicate better attitudes towards PMI. Comparing the mean (SD) ATPMI domain scores in 2015 study and 2023 study, it was observed that social distancing (8.07 (2.95) vs. 7.19 (2.84)), social restrictiveness (7.21 (2.90) vs. 6.22 (2.64)), and prejudice and misconception (15.36 (4.09) vs. 14.06 (3.86)) scores were lower in 2023 than in 2015, while the mean tolerance/support for community care score (14.81 (3.91) vs. 15.23 (4.11)) was higher than that observed in 2015 (Table 2). Comparison between the two studies revealed a significant improvement across all four domains of the scale (*p* < 0.001) in the intervening eight years.

**Table 2:** Comparison of mean scores of ATPMI between 2015 and 2023

	Mind Matters 2015		Mind Matters 2023		*p*-value
	Weighted mean	SD	Weighted mean	SD	
Social distancing (Range: 3–15)	8.07	2.95	7.19	2.84	**< 0.001**
Tolerance/support for community care (Range: 9–45)	14.81	3.91	15.23	4.11	**< 0.001**
Social restrictiveness (Range: 3–15)	7.21	2.90	6.22	2.64	**< 0.001**
Prejudice and misconception (Range: 5–25)	15.36	4.09	14.06	3.86	**< 0.001**

Note: p-values were derived using linear regression with ATPMI scores as outcomes and timepoint as independent variable

**Table 3:** Sociodemographic correlates associated with the four domains of ATPMI in 2023

Variables		Social distancing				Tolerance/support for community care				Social restrictiveness				Prejudice and misconception			
		β	95% CI		*p*	β	95% CI		*p*	β	95% CI		*p*	β	95% CI		*p*
Age groups	18–34	Reference				Reference				Reference				Reference			
	35–49	0.74	0.38	1.09	**< 0.001**	0.09	-0.39	0.56	0.714	0.82	0.54	1.09	**< 0.001**	1.02	0.60	1.44	**< 0.001**
	50–67	1.21	0.82	1.59	**< 0.001**	0.09	-0.43	0.62	0.723	1.50	1.17	1.83	**< 0.001**	1.55	1.08	2.02	**< 0.001**
Gender	Male	Reference				Reference				Reference				Reference			
	Female	-0.21	-0.46	0.04	0.099	0.37	0.02	0.73	**0.038**	-0.45	-0.67	-0.24	**< 0.001**	-0.56	-0.86	-0.26	**< 0.001**
Ethnicity	Chinese	Reference				Reference				Reference				Reference			
	Malay	0.11	-0.13	0.35	0.365	0.07	-0.28	0.42	0.689	-0.16	-0.37	0.05	0.136	0.87	0.58	1.16	**< 0.001**
	Indian	-0.46	-0.69	-0.23	**< 0.001**	0.37	0.04	0.70	**0.029**	-0.36	-0.56	-0.16	**0.001**	0.82	0.54	1.11	**< 0.001**
Marital Status	Currently married	Reference				Reference				Reference				Reference			
	Never married	-0.58	-0.90	-0.26	**< 0.001**	0.44	-0.01	0.90	0.054	-0.44	-0.71	-0.17	**0.001**	-0.55	-0.95	-0.16	**0.006**
	Separated/ Widowed/Divorced	-0.24	-0.77	0.28	0.359	0.03	-0.77	0.83	0.946	-0.29	-0.78	0.20	0.243	0.33	-0.46	1.11	0.417
Education	University and above	Reference				Reference				Reference				Reference			
	Primary and below	0.09	-0.49	0.67	0.762	-2.92	-3.73	-2.11	**< 0.001**	2.12	1.52	2.71	**< 0.001**	4.14	3.44	4.85	**< 0.001**
	Secondary	-0.02	-0.41	0.38	0.932	-1.94	-2.51	-1.37	**< 0.001**	0.81	0.45	1.17	**< 0.001**	2.66	2.18	3.14	**< 0.001**
	Pre-university	-0.21	-0.52	0.10	0.187	-0.52	-0.96	-0.09	**0.019**	0.16	-0.11	0.43	0.241	1.54	1.16	1.92	**< 0.001**
Employment status	Currently employed	Reference				Reference				Reference				Reference			
	Unemployed	-0.18	-0.79	0.43	0.553	-0.06	-0.88	0.77	0.895	0.10	-0.42	0.63	0.698	-0.06	-0.87	0.75	0.664
	Economically inactive	-0.20	-0.62	0.23	0.365	0.23	-0.43	0.89	0.496	-0.16	-0.58	0.26	0.461	-0.32	-0.88	0.23	0.282
Personal income	Above SGD6000	Reference				Reference				Reference				Reference			
	Below SGD2000	0.53	0.07	0.99	**0.024**	-0.67	-1.36	0.01	0.054	0.71	0.28	1.14	**0.001**	1.02	0.46	1.58	**0.001**
	2000 to SGD5999	0.34	-0.01	0.69	0.058	-0.71	-1.20	-0.22	**0.004**	0.52	0.22	0.82	**0.001**	0.94	0.51	1.36	**< 0.001**

### Sociodemographic correlates of ATPMI

Older adults were found to have higher social distancing (35–49: β = 0.74, *p* < 0.001, 50–67: β = 1.21, *p* < 0.001), social restrictiveness (35–49: β = 0.82, *p* < 0.001, 50–67: β = 1.50, *p* < 0.001), and prejudice and misconception (35–49: β = 1.02, *p* < 0.01, 50–67: β = 1.55, *p* < 0.001) scores as compared to younger adults aged 18–34. No associations were found between the age groups for tolerance/support for community care. Females were associated with lower social restrictiveness (β=-0.45, *p* < 0.001), prejudice and misconception (β=-0.56, *p* < 0.001) and higher tolerance/support for community care (β = 0.37, *p* = 0.038) scores compared to males. As compared to Chinese ethnicity, both Malay (β = 0.87, *p* < 0.001) and Indian (β = 0.25, *p* < 0.001) ethnicities were more likely to have higher prejudice and misconception towards PMI. However, respondents of Indian ethnicity were also found to have lower social distancing (β=-0.46, *p* < 0.001), social restrictiveness (β=-0.37, *p* = 0.001) and greater tolerance/support for community care (β = 0.37, *p* = 0.029) as compared to those of Chinese ethnicity. Respondents who were never married were more likely to have lower social distancing (β=-0.58, *p* < 0.001), lower social restrictiveness (β=-0.44, *p* = 0.001) and lower prejudice and misconception (β=-0.55, *p* = 0.006) as compared to those who were currently married.

As compared to respondents with a bachelor’s degree and above, those with lower educational levels have lower tolerance/support for community care (primary: β=-2.92, *p* < 0.001, secondary: β=-1.94, *p* < 0.001, pre-university: β=-0.52, *p* = 0.019), greater social restrictiveness (primary: β = 2.12, *p* < 0.001 secondary: β = 0.81, *p* < 0.001), and greater prejudice and misconception (primary: β = 4.14, *p* < 0.001 secondary: β = 2.66, *p* < 0.001 pre-university: β = 1.54, *p* < 0.001). Interestingly, this pattern was not seen for social distancing. Additionally, the current study did not find any associations between employment status and any of the domains. For personal income, compared to those earning more than SGD$6000, respondents in the lower income groups were associated with greater social distancing (Below SGD$2000: β = 0.53, *p* = 0.024), social restrictiveness (Below SGD$2000: β = 0.71, *p* = 0.001, SGD$2000–5999: β = 0.52, *p* = 0.001), and prejudice and misconception (Below SGD$2000: β = 0.91, *p* = 0.001, SGD$2000–5999: β = 0.88, *p* < 0.001). Furthermore, as compared to those earning more than SGD$6000, respondents earning between SGD$2000 to $5999 displayed lower tolerance/support for community care (β=-0.68, *p* = 0.007). Detailed results of the sociodemographic correlates are presented in Table 3. 

## Discussion

The present study investigated whether attitudes towards PMI across four factors (social distancing, tolerance/support for community care, social restrictiveness, and prejudice and misconception) differed between 2015 and 2023 and examined the sociodemographic correlates associated with these attitudes in 2023. The findings of the study reflect a positive shift in attitudes, aligning with evidence from mental health professionals [30, 31]. These improved attitudes are important as there is evidence that people’s intention to seek help is positively associated with better public support [4, 34]. However, it is important to note that whilst overall attitudes have improved, scores for tolerance/support for community care remain relatively low, indicating that the public remains apprehensive about community care. Other studies have postulated that this may be attributed to the “not in my backyard” phenomenon, where individuals oppose the inclusion of mental health services in their neighbourhood, even if they support the need for such services elsewhere [35,36,37]. This reluctance could stem from various concerns, such as physical safety or perceiving a more critical need for services for other groups in the community [35,38]. This lack of support and tolerance for community care can lead to lower quality, accessibility, and availability of services that PMI might need, highlighting the importance of fostering support among the public [38].

The sociodemographic characteristics associated with attitudes did not differ much in 2023 compared to 2015. Consistent with Mind Matters 2015, the 2023 study revealed that older individuals, males, and individuals with lower educational levels had poorer attitudes towards PMI. One possible explanation for younger adults having better attitudes is their greater exposure to mental health information through social media platforms leveraged by many local campaigns. A 2020 study found that social distancing tendencies increase with age, possibly explained by the ‘impressionable years model’, which proposes that attitudes formed in younger years remain stable through adulthood [39]. This could explain why older adults are still associated with worse attitudes [39]. Corroborating this notion, a meta-analysis conducted to explore changes in public stigma among respondents from 14 different countries revealed that current mental health educational interventions are generally more effective with adolescents as compared to their older counterparts [40].

Furthermore, consistent with present findings, other studies have also reported that females tend to hold more positive attitudes toward PMI than males across different measures [41, 42]. Cultural and gender norms strongly influence perceptions of what topics are appropriate for discussion and the types of support that are considered acceptable [43]. Cross-cultural perspectives suggest that mental health-related shame in many Asian societies stems from enduring cultural and religious beliefs that prioritise family honour and view mental illness as a personal or moral failing [44,45,46]. When these beliefs intersect with traditional masculine norms that emphasise strength, emotional restraint, and responsibility as the family provider, mental health difficulties are often trivialised as personal weakness, leading to greater negative attitudes and reduced empathy towards PMI [44,47,48,49]. Previous studies have also indicated that females are more likely to seek support for mental health conditions despite similar prevalence rates among both genders, possibly due to sociocultural expectations that portray men as strong and self-reliant [43,50]. Collectively, these differences might help explain why females have better attitudes towards PMI than males.

The findings also indicated that while Indian and Malay respondents exhibited higher levels of prejudice and misconceptions, Indian respondents demonstrated lower social distancing and restrictiveness, as well as greater tolerance/support for community care compared to Chinese respondents, patterns consistent with those observed in 2015. Cultural frameworks appear to shape the expression and sources of negative attitudes differently across ethnic groups. Tan and colleagues (2020) noted that etiological beliefs surrounding mental illness vary between different cultures in Singapore, which may contribute to distinct forms of stigma [3]. In Indian and Malay communities, explanations such as religion, retribution, and supernatural elements are often associated with stigma [51,52,53]. In contrast, within Chinese societies, stigma is closely tied to the cultural construct of “loss of face”, a reflection of social status and moral reputation [54]. Another local study has shown that although PMI are frequently perceived as dangerous and unpredictable by Indian respondents, they tend to be more accepting and willing to engage with them compared to their Chinese counterparts [25]. Similar findings from India suggest that despite persistence of stigma, PMI often receive support from their families and communities [55,56]. However, more research is needed to clarify how these etiological beliefs translate into specific manifestations of attitudes.

In 2023, respondents who were never married scored better in three domains of attitudes as compared to one (prejudice and misconception) in 2015. Marital status is typically associated with age, as younger adults are more likely to fall under the ‘never married’ category [57]. In our sample, the majority of respondents in the “never married” category also fell within the “18–34” age category, further supporting the observed age-related differences in attitudes. Additionally, apart from the consistent finding that those earning less were more likely to hold more prejudice and misconceptions, the 2023 study revealed that those earning less also had higher social distancing and restrictiveness and lower tolerance/support for community care. These changes in trends could plausibly suggest that improvements in attitudes were mainly amongst those in higher income groups. These individuals are more likely to have better access to information regarding mental health and the importance of social inclusivity for PMI. A study conducted in the United States highlighted that individuals in higher income groups were more likely to perceive a more unsupportive environment for PMI [58]. If this holds for the present study, it could be speculated that positive attitudes towards community care among those in higher income groups reflect their support for creating a more inclusive and supportive environment for PMI.

The consistent pattern of poorer attitudes among older adults, males, and individuals with lower socioeconomic status suggests that recent destigmatisation efforts have not effectively reached or influenced certain vulnerable groups, and measures should be taken to increase accessibility. Multi-pronged strategies that combine education with personal contact and target specific community settings are needed. Culturally sensitive, community-level interventions are essential, and the persistent link between ethnicity and attitudes underscores the importance of engaging key religious and community leaders [12,59,60,61,62]. Additionally, interventions that incorporate contact with PMI alongside education serve as an effective strategy directed towards positive changes in attitudes and fostering empathy towards PMI [41, 63, 64]. Current public initiatives in Singapore typically focus on education, whereas opportunities for contact are lacking. Some beneficial contact-based interventions have been implemented for specific subgroups. For example, an anti-stigma intervention implemented for local university students that involved both education and contact revealed promising short-term improvements in community attitudes towards mental illness among respondents [65]. It would be beneficial to extend access to these interventions for other subgroups, tailoring them to specific needs (e.g., delivering them in different local languages). Beyond these, integrating mental health care into general or primary healthcare settings is advantageous as it helps reduce stigma on top of improving access to care, especially for older adults who may already often use these facilities for physical conditions [59,66]. When mental health care is accessible within familiar healthcare settings, it helps normalise it.

The results of this study should be viewed in light of the following limitations. Firstly, although the questionnaire was administered through an interviewer to ensure response quality, respondents might have provided socially desirable responses as the topic pertained to attitudes [67]. Secondly, the present study used the umbrella term ‘mental illness’ when investigating stigma, however, individuals may hold different attitudes towards different mental illnesses [68]. Thus, these responses might have been affected by how the respondent interpreted the term, suggesting some levels of inconsistency. Notwithstanding these, the present study holds several strengths, including a large sample size generalisable to the general population, the use of multiple local languages ensuring inclusivity and the consistent methodology between the 2015 and 2023 surveys, ensuring that valid comparisons and conclusions can be made regarding changes in attitudes in the general population.

The present study provides a nationally representative update regarding public attitudes towards PMI in Singapore, comparing data between 2015 and 2023. The findings indicate modest improvement in attitudes over time, but relatively poor attitude scores for tolerance/support for community care, and the persistence of subgroups associated with worse attitudes highlights the need for improving and strategizing on our current mental health initiatives. These results extend the empirical literature by confirming that, despite broader societal shifts and destigmatisation efforts, certain subgroups remain vulnerable to entrenched stigma. Compared to prior studies primarily conducted in Western contexts or smaller Asian samples, this study offers a comprehensive view of public perceptions within a multiethnic Asian society.

In future, it would be beneficial to delve deeper and investigate the underlying causes for poorer attitudes towards PMI among subgroups for the development of targeted initiatives. Bradbury (2020) suggested a longitudinal approach in investigating attitude changes over time within-subjects to have a deeper understanding of relevant life-course determinants [41]. Additionally, it is also recommended that future studies include the degree of previous contact with PMI, as prior evidence consistently shows that personal contact is one of the strongest predictors of reduced stigma [34,69,70]. Incorporating this variable would provide a more comprehensive understanding of the factors shaping public attitudes and help identify potential leverage points of interventions. Furthermore, future studies can also examine whether improved attitudes translate into tangible behavioural changes, such as an increase in support for mental health policies, reduced treatment gaps, or better support in workplaces. Continuous investigation of attitudes in the nation would be beneficial in understanding emerging trends and further strategizing and improving mental health initiatives.

## Data Availability

The datasets presented in this article are not readily available because the authors’ government law and institution only permits the sharing of human participant data with researchers with whom they have a Research Collaboration Agreement (RCA). However, data sharing with clear research purposes can be made available upon request. Requests to access the datasets should be directed to mythily@nhghealth.com.sg.
